# Surveillance of Humans Exposed to the Potentially Rabid Animals and Rabies Post-Exposure Prophylaxis in Split-Dalmatia County, Croatia, 2015–2024

**DOI:** 10.3390/medicina61122119

**Published:** 2025-11-28

**Authors:** Anamarija Jurčev Savičević, Josip Buzov, Inga Vučica, Ivana Marasović Šušnjara, Nora Josipa Savičević

**Affiliations:** 1 Teaching Public Health Institute of Split-Dalmatia County, Vukovarska 46, 21 000 Split, Croatia; 2 School of Medicine, University of Split, Šoltanska 2A, 21 000 Split, Croatia; 3 Faculty of Health Sciences, University of Split, R. Boškovića 35, 21 000 Split, Croatia; 4 Agram Special Hospital, Varaždinska 54, 21 000 Split, Croatia; 5 Family Medicine Office, Jurja Šižgorića 20, 21 000 Split, Croatia

**Keywords:** rabies prevention, rabies, post-exposure prophylaxis, Croatia

## Abstract

*Background and Objectives*: Although preventable, rabies represents a significant public health problem. An important part of prevention is the surveillance of people exposed to potentially rabid animals, carried out in the anti-rabies clinics of all public health institutes in Croatia. We aimed to analyze the burden of human animal-bite injuries, patient/biting animal characteristics, and the uptake of anti-rabies post-exposure prophylaxis (PEP). *Material and Methods*: This retrospective study used medical records data ranging from 2015 to 2024 for all patients in the anti-rabies clinics in Split-Dalmatia County, Croatia. *Results*: A total of 4105 patients reported contact with a potentially rabid animal. The majority of examined people (52.6%) were working-aged adults (20–60 years). The largest proportion of reported injuries were recorded on the lower limbs (34.9%) and hands/fingers (32.3%). No contact with a proven rabid animal was recorded. PEP was received by 37.7% of those examined. Although dog exposure (68.9%) most frequently led to post-exposure care-seeking, PEP was most common after rodent (91.2%) and bat (87.5%) exposures. *Conclusions*: Improving public health education is the most effective method of preventing dog bites and thus reducing bite injuries. Promoting responsible dog ownership and behavior around animals, as well as avoiding contact with unfamiliar animals, would likely reduce the need for PEP. The results of this study can also be used in planning health resources, primarily the availability of rabies vaccine and immunoglobulin. In addition, they emphasize the importance of rabies prevention and the continued implementation of all preventive measures in collaboration between the human and animal health sectors. This research may be useful to future public health policies for the control of zoonotic infectious diseases, especially from a “One Health” perspective.

## 1. Introduction

Rabies is an acute infectious disease caused by viruses belonging to the *Lyssavirus* genus, *Rhabdoviridae* family. This zoonotic disease affects the central nervous system of mammals, including dogs, cats, livestock, wildlife, and humans, causing severe encephalopathy and generalized paresis [1,2,3].

The most frequent virus that causes human rabies is *Rabies Lyssavirus*, which commonly spreads to people through bites or scratches from infected mammals, usually infected dogs. There is no evidence of human-to-human transmission, except in the extremely rare cases of infected tissue and organ transplantation [3]. Although human rabies has a very high fatality rate within five to seven days after the onset of symptoms, there are some reports of survival of patients with clinical rabies [4,5].

Despite being entirely preventable in both humans and animals, rabies remains a significant public health problem, with an estimated 59,000 human deaths annually, particularly in poor rural communities in Africa and Asia [6]. According to the European Centre for Disease Prevention and Control, five cases of human lyssavirus infection were reported from 2019 to 2023 in the European Union, four cases of which were imported. One EU-acquired human lyssavirus infection was caused by European bat lyssavirus 1. In the same period, a total of 425 wild and domestic animals, including bats, had positive results of lyssaviruses. The main rabies carrier in Europe is the red fox [7].

Croatia is a European Union country, and the last case of human rabies was recorded in 1964. Since then, only two imported cases of human rabies were notified, one in 1989 and one in 1996, both in patients from Bosnia and Herzegovina, a neighboring country [8]. The last case of a rabid animal (a red fox) was recorded in 2014, with a continuous decrease in the number of rabid animals since 2008 [9].

However, the public health hazards related to rabies must not be neglected in Croatia due to the presence of rabies in Europe. Preventive measures in Croatia include mandatory vaccination of dogs and recommended vaccination of cats and ferrets (lat. *Mustela putorius furo*), educating the population on animal behavior and bite prevention, and human rabies prophylaxis [10]. Rabies vaccination is used for pre-exposure prophylaxis for populations at high risk and for post-exposure prophylaxis along with rabies immunoglobulin, if indicated [11]. When possible, a 10-day veterinary monitoring period should be applied to the animal that was the reason for post-exposure care-seeking. Surveillance of people exposed to potentially rabid animals is provided in all 21 county institutes of public health and the Croatian Institute of Public Health [12].

In the following sections, we will provide an overview of rabies post-exposure surveillance, including the gender and age structure of examined persons, localization of injuries, epidemiological groups of animals, animal species, and rabies post-exposure prophylaxis in Split-Dalmatia County, the second-largest and most inhabited Croatian county. This research may be useful for the creation of public health policies for the control of zoonotic infectious diseases, especially from a “One Health” perspective [13].

## 2. Materials and Methods

All the participants in this retrospective study were persons who reported to the anti-rabies clinics (ARCs) of the Teaching Institute for Public Health of the Split-Dalmatia County (TIPH) due to potential rabies exposure from 2015 to 2024, including the clinic at headquarters in Split and all branch clinics of the institute throughout the county (Brač, Hvar, Trogir, Vrgorac, Makarska, Sinj, Omiš, Imotski).

For research purposes, the patients were divided into the following age groups: 0–10, 11–20, 21–30, 31–40, 41–50, 51–60, and 60+ years.

Animals were divided into the following four groups: A—confirmed rabid animal or contact with proven rabid animal or contaminated material; B—animal suspected of rabies due to unusual behavior; C—unknown, dead, stray, killed, or wild animal; and D—animal that remained healthy after 10 days of observation.

Rabies post-exposure prophylaxis (PEP) includes a rabies vaccine or a combination of rabies vaccine and human rabies immunoglobulin.

The research was approved by the Ethics Committee of TIPH of the Split-Dalmatia County at its 28th session held on 10 January 2025.

## 3. Results

According to the ARCs in Split-Dalmatia County records, a total of 4105 people were examined due to potential rabies exposure between 2015 and 2024; 2079 (50.7%) men and 2020 (49.3%) women. Gender and age data were missing for six people. Majority of the patients (18.3%) were in the age group 60+ years, while children up to 10 years accounted for 15.7% (Table 1).

A total of 4096 patients reported animal injuries, while possible contact without a wound was recorded in 9 persons (e.g., a bat flying into the bedroom). The largest share of reported injuries was recorded on the lower limbs (34.9%), followed by the hands and fingers (32.3%), upper limbs (18.1%), and head and neck (6.3%). Injuries in more than one place were recorded in 4.9% of persons, and the trunk was injured in 3.3% of examined persons (Table 2).

Regarding the animal group, it should be noted that all the patients were examined for exposure to animals in groups C (1652; 40.2%) and D (2543; 59.8%). No exposures were examined in group A or group B. A majority of the examined patients (2866; 69.8%) were in contact with a dog, followed by contact with a cat (1017; 24.7%). More than 100 (2.49%) patients reported injuries from rodents, while 24 (0.58%) patients reported contact with a bat. Contact with ungulates (0.34%), foxes (0.19%), and ruminants (0.10%) was rarely reported. Other animals were reported in 70 (1.71%) patients (Table 3).

Out of a total of 4105 people who reported to the ARCs, 62.3% of them were examined with no prescribed PEP. As can be seen from Table 4, 1376 (33.5%) patients received PEP due to exposure to animals from group C, and 172 (4.2%) from group D. As a part of post-exposure prophylaxis, 1437 (35%) persons received the vaccine alone, while 111 (2.7%) received, in addition to the vaccine, human rabies immunoglobulin. In persons exposed to a group C animal, 1280 (31.2%) were vaccinated, while 96 (2.3%) received the vaccine and HRIG. A total of 172 persons with exposure in category D received PEP; 157 (3.8%) of them received the vaccine only, and 15 (0.04%) received both the vaccine and HRIG.

Of the 1548 patients who received PEP, 605 (39.1%) had injuries to the hand and fingers, 496 (32%) to the lower limbs, and 239 (15.4%) to the upper limbs. Injuries in multiple places were recorded in 88 (5.7%), in the head and neck in 81 (5.2%), and in the trunk in 32 (2.1%) patients who received PEP. Due to possible contact without a wound, seven patients (0.05%) received PEP (Table 5).

Regarding the type of animal and total number of patients who received PEP, the most common were dogs in 830 (53.6%) cases, cats in 552 (35.6%), and rodents in 93 (6%). Bats were recorded in 21 (1.4%) patients with PEP, ungulates in 7 (0.05%), foxes in 4 (0.3%), and ruminants in 2 (0.1%). Other animals were represented as a reason for PEP in 37 (2.4%) patients.

If we take into account the total number of potential rabies exposures and the percentage that required prophylaxis, rodent exposures were the most frequent (91.2%), followed by bat exposures (87.5%) and cat exposure (54.3%). Ungulates, foxes, and ruminants were presented with 50% prophylaxis in each category. The least frequent exposure that required prophylaxis was dog exposure (29%) (Table 6).

## 4. Discussion

In this research, data on all persons (4105) examined for contact with animals suspected of rabies in ARCs in Split-Dalmatia County in the period from 2015 to 2024 were analyzed.

Gender distribution indicates that exposure to a potentially infected animal is equally present in both sexes. Other studies found higher prevalence of males among post-exposure care seekers, which was explained by occupational or behavioral factors influencing risk exposure [14,15,16]. Regarding age distribution, working-aged adults (20–60 years) are the most frequently exposed to potentially rabid animals, as presented elsewhere [12,14].

The localization of injuries is not random, but clearly concentrated in certain anatomical regions. The largest number of injuries was registered on the lower limbs, followed by the hands and fingers, which together accounted for almost two-thirds of all injuries. Such a distribution of injuries is not unexpected; legs are a part of the human body that are easily accessible to animals, while injuries to the hand and finger mostly occur when separating animals in collision, touching animals, feeding, or defending against animals. Such distribution of injuries was also shown in other studies [12,14,15].

In one study referring to the previous fifteen-year period from 2000 to 2014, among all persons examined in the same ARCs as this study, 4.7% of patients were in contact with a proven rabid animal and 1.4% were in contact with an animal suspected of rabies [17]. This indicates an improvement in rabies control in Croatia. The number of rabid animals showed a continuous decrease since 2008, with the last rabid animal reported in 2014 [9]. This is a direct consequence of oral rabies vaccination targeting red foxes and golden jackals (throwing baits from airplanes). It has been systematically carried out twice a year throughout Croatia since 2011 [8]. In addition, mandatory vaccination of dogs and recommended vaccination of cats and ferrets remain in place.

Surveillance of persons due to potential rabies exposure, as well as pre-exposure prophylaxis, is widely available in Croatia, further reducing the risk of disease in humans. In addition, veterinarians monitor animals for ten days upon notification from ARCs, mostly dogs and other pets or farmed mammals, and report back their health status. In cases of suspected rabies or failed monitoring, they immediately notify the ARCs for further action.

Due to successful Croatian program for rabies eradication, the European Commission officially declared Croatia as a rabies-free country in 2021 [18].

Nevertheless, a significant number of patients who had contact with an unknown, dead, stray, or wild animal, or an animal of unknown health status, emphasizes the need to improve the mechanisms of animal identification and monitoring. According to the Animal Health Act, all dogs must be marked with an injectable transponder no later than 90 days after the day they were born. For cats and ferrets, marking with an injectable transponder is recommended; it is performed at the owner’s request, and is mandatory before moving to another country. All animals marked in this way are registered in the Pet Register in the computer database, thus facilitating their monitoring [10].

The most common localization of injury for which people are protected by PEP, the hand/fingers and lower limbs, corresponds to the most common localization of injuries in general. Taking into account the high prevalence of injuries on the hands and lower limbs, it can be concluded that educating the population about bite prevention and proper behavior towards an animal can play an important role in reducing the number of incidents that require prophylaxis. This especially applies to situations involving contact with unknown animals or strays. Also, people engaged in outdoor activities (staying in caves, camping, hunting, etc.) should be educated about avoiding contact with wild animals, and the same is suggested for travelers visiting countries where rabies is more common or endemic.

Eastern Europe, for example, regularly has a higher number of recorded rabid animals than Western and Central European countries [19,20]. The latest reported human rabies case was in July 2025; a man from Romania died after being bitten by a rabid stray dog [21]. In addition, the war in Ukraine further threatens the efforts made by Western European countries to eliminate rabies. Namely, Ukraine is a country that has been among the highest-ranking European countries in terms of the number of rabid animals for years. The war that started in 2022 led to increase in the number of human bites from both domestic and wild animals, as well as laboratory-confirmed cases of rabies in animals in Ukraine. In a nine-month period during 2023, the number of rabies prophylaxis due to animal injuries increased by more than 40%. It seems that the huge population of homeless dogs (more than one million) and the increasing competition for food among animals increase the risk of conflict between animals and humans. It was noted that efforts for oral vaccination of foxes and regular rabies vaccination were also affected by the war [22]. Cases of rabies among animals were recorded sporadically in Croatia’s neighboring countries. Hungary had 15 cases of rabies of autochthonous origin in 2023 in animals excluding bats [7]. Croatia has a long border with Bosnia and Herzegovina. Two imported cases of rabies in humans, recorded in 1989 and 1995, originated from Bosnia and Herzegovina [12]. In the last five years, sporadic cases of rabid animals were reported in Bosnia and Herzegovina and Serbia [23,24].

Although dog exposure (68.9%) most frequently led to post-exposure care, rabies post-exposure prophylaxis was most common after rodent (91.2%) and bat (87.5%) exposure. Documented cases of rabies in rodents are rare, indicating the need for careful assessment before administering PEP [25]. Regarding bats, one Croatian study reported that the bat population in Croatia had a confirmed presence of *Lyssavirus*, although with no positive bat brain isolates or bat-related deaths in humans [8]. Human rabies cases upon exposure to bats are well documented [7,26].

From 2024, the Croatian program of immunization, seroprophylaxis, and chemoprophylaxis, specifies in detail the need of prophylaxis upon exposure to bats. If a person had uncertain physical contact with the saliva of a live bat, but there is a possibility that this contact happened (e.g., touching a bat without gloves, a bat that became entangled in hair, a person who does not remember with certainty the kind of contact they had with a bat, or sleeping in a room where a bat was), a rabies vaccine should be given, while in the case of injuries caused by bats, the patient should receive a rabies vaccine and immunoglobulin [11]. However, the indications for rabies PEP have been narrowed in general due to the fact that no terrestrial rabies has been detected in the past ten years. The category of provoked bite is singled out as one where PEP is not required, for example, if a person separates their dog from someone else’s, or touches or feeds an unknown animal that has an owner. This does not refer to a direct animal attack on a human [11].

Although Croatia has been declared a rabies-free country, maintaining this status depends on the continuous implementation of all preventive measures against rabies, as well as on the burden of rabies in neighboring countries, since animals, especially foxes, have a large range of movement. Another threat comes from infected bats, which, unlike foxes and domestic animals, are not easy to vaccinate. Trade and travel also carry a constant risk of reintroduction of rabies.

A limitation of this research is that we cannot extract data related to stray animals to influence policy and practice related to the control of stray animals. The strength of this research is the respectable research period, the large sample, and the study location, the second-largest and most populated Croatian county. Since rabies prevention measures are the same throughout the country, we believe that these data are representative for Croatia in general.

## 5. Conclusions

Although there have been no rabid animals in Croatia for a decade, the epizootic and epidemiological situation in neighboring and other European countries indicates that caution and consistent adherence to measures to prevent rabies are still needed.

The results of this research can be used in the evaluation of rabies post-exposure prophylaxis and in the planning of health resources, primarily the availability of rabies vaccine and immunoglobulin. In addition, it emphasizes the importance of rabies prevention at the level of humans and animals, as effective public health strategies in collaboration between the human and animal health sectors. This research may be useful for future public health policies for the control of zoonotic infectious disease, especially from the “One Health” perspective.

## Figures and Tables

**Table 1:** Distribution of patients examined in anti-rabies clinics in Split-Dalmatia County due to potential rabies exposure in the period 2015–2024 by gender and age groups.

Age Groups (Years)	Males	Females	Total	%
0–10	345	298	643	15.7
11–20	294	258	552	13.4
21–30	304	310	614	15
31–40	266	227	493	12
41–50	254	257	511	12.5
51–60	246	289	535	13.1
60+	370	381	751	18.3
Total	2079	2020	4099 *	100

* gender and age data missing for six patients.

**Table 2:** Distribution of patients examined in anti-rabies clinics in Split-Dalmatia County clinics due to potential rabies exposure in the period 2015–2024 by localization of injury.

Localization of the Injury	Total	%
Lower limbs	1431	34.9
Hand and fingers	1325	32.3
Upper limbs	742	18.1
Head and neck	259	6.3
Multiple	203	4.9
Trunk	136	3.3
Possible contact with no wound	9	0.2
Total	4105	100

**Table 3:** Distribution of patients examined in anti-rabies clinics in Split-Dalmatia County due to potential rabies exposure in the period 2015–2024 by animal species and animal group *.

Animal	Group C	Group D	Total	%
Dog	859	2007	2866	69.8
Cat	610	407	1017	24.7
Rodents	89	13	102	2.49
Bat	24	0	24	0.58
Ungulates	1	13	14	0.34
Fox	8	0	8	0.19
Ruminants	1	3	4	0.10
Other animals †	60	10	70	1.71
Total	1652	2453	4105	100

* there were no animals in group A and group B. † pig, boar, meerkat, marten, chicken, iguana, hedgehog, monkey, crow, rabbit, and unknown animals.

**Table 4:** Distribution of patients examined in anti-rabies clinics in Split-Dalmatia County due to potential rabies exposure in the period 2015–2024, by rabies post-exposure prophylaxis and animal group.

Group of Animals	Number of Participants	Rabies Post-Exposure Prophylaxis		
		Vaccine	Vaccine + HRIG *	Total
A	0	0	0	0
B	0	0	0	0
C	1652	1280	96	1376
D	2453	157	15	172
Total	4105	1437	111	1548

* human rabies immunoglobulin.

**Table 5:** Distribution of rabies post-exposure prophylaxis (vaccine + HRIG *) in anti-rabies clinics in Split-Dalmatia County in the period 2015–2024 by localization of injury.

Localization of the Injury	Rabies Post-Exposure Prophylaxis	
	Total	%
Hand and fingers	605	39.1
Lower limbs	496	32.0
Upper limbs	239	15.4
Multiple	88	5.7
Head and neck	81	5.2
Trunk	32	2.1
Possible contact with no wound	7	0.5
Total	1548	100

* human rabies immunoglobulin.

**Table 6:** Distribution of rabies post-exposure prophylaxis (vaccine + HRIG *) in anti-rabies clinics in Split-Dalmatia County in the period from 2015 to 2024 by animal species.

Animal	Rabies Post-Exposure Prophylaxis		Potential Rabies Exposure That Required Prophylaxis
	Total	%	%
Dog	830	53.6	29
Cat	552	35.6	54.3
Rodents	93	6	91.2
Bat	21	1.4	87.5
Ungulates	7	0.5	50
Fox	4	0.3	50
Ruminants	2	0.1	50
Other animals †	37	2.4	55.7
Total	1548	100	

* human rabies immunoglobulin. † pig, boar, meerkat, marten, chicken, hedgehog, monkey, crow, rabbit, and unknown animals.

## Data Availability

The raw data supporting the conclusions of this article will be made available by the authors on request.

## Abbreviations

The following abbreviations are used in this manuscript:

ARCs	Anti-Rabies Clinics
HRIG	Human Rabies Immunoglobulin
PEP	Post-exposure Prophylaxis
TIPH	Teaching Institute for Public Health of the Split-Dalmatia County
